# Endogenous Cannabinoid Production in the Rat Female Reproductive Tract Is Regulated by Changes in the Hormonal Milieu

**DOI:** 10.3390/ph4060933

**Published:** 2011-06-23

**Authors:** Heather B. Bradshaw, Cassandra Allard

**Affiliations:** 1 Department of Psychological and Brain Sciences, Bloomington, IN, 47405, USA; 2 The Kinsey Institute of Sex, Gender, and Reproduction at Indiana University, Bloomington, IN, 47405, USA

**Keywords:** endocannabinoid, female, uterus, ovary, estrous

## Abstract

The endogenous cannabinoid (eCB) system is emerging as an important component of female reproductive tract physiology. The eCBs anandamide (AEA), 2-arachidonoyl glycerol (2-AG), and *N*-arachidonoyl glycine (NAGly) were measured in the rat reproductive tract at five time points in the four-day estrous cycle, in acyclic retired breeders (RB), after ovariectomy (OVX), OVX + estrogen (E2), OVX + progesterone (P4), or OVX with E2+P4. eCBs were measured in the uterus, uterine adipose, ovaries, and ovarian adipose using HPLC/MS/MS. Levels of AEA, 2-AG, and NAGly were highest in the estrus phase of the estrous cycle in the uterus, whereas, only NAGly had differences in production in the ovaries across the cycle. All eCBs were lower in RB ovaries; however, the production of eCBs in the uterus of RB and OVX groups was more varied with NAGly showing the lowest levels of production in these groups. Levels of AEA in uterine fat were significantly higher or equivalent to levels in the uterus. However, levels of 2-AG and NAGly were dramatically lower in uterine fat verses the organ. Ovarian fat had significantly lower levels of all three eCBs. These data provide evidence that the hormonal milieu plays a significant and complex role in the production of eCBs in the female rat reproductive tract.

## 1. Introduction

Endocannabinoid ligands (eCB) were named as the endogenous counterpart of the biologically active phytocannabinoids found in the cannabis plant. However, it is the eCBs that evolved to activate endogenous receptors, which allows the phytocannabinoids to mimic them. At the core of the eCB receptor-ligand system are two G-protein coupled receptors (GPCRs; CB_1_ and CB_2_) [1], which are activated by the endogenous lipophilic ligands, 2-arachidonoyl glycerol (2-AG) [2] and arachidonoyl ethanolamine (AEA) [3]. When CB_1_ or CB_2_ is activated the cumulative result is typically a decrease in cellular excitability or transmitter release. However, activation of these receptors modulate multiple levels of intracellular signaling [1].

AEA is primarily degraded by fatty-acid amide hydrolase (FAAH) and this enzyme serves to modulate eCB activity [4]. An endogenous metabolite of AEA, *N*-arachidonoylglycine (NAGly) [5] has antinociceptive and anti-inflammatory activity [6]. We recently demonstrated that AEA is metabolized to NAGly through two distinct pathways [5]. The metabolism of AEA to NAGly progresses through: 1) a FAAH-dependent pathway and 2) a FAAH-independent alcohol dehydrogenase pathway that oxidizes ethanolamine to glycine. NAGly does not, however, activate the CB_1_ or CB_2_ receptors [7], but rather activates two different orphan GPCRs (GPR18 [8,9] and GPR 92 [10]), suggesting NAGly, GPR18, and GPR92 are additional members of a broader eCB system.

The eCB system plays an important role in reproduction [11-14]. A brief review of current findings suggests that, given the ubiquitous nature of the eCB system and the myriad of systems involved in reproduction, the evolution of the intersection of these two systems regulates: 1) the HPA axis, 2) sperm and ova motility, 3) implantation, 4) sexual behavior, and 5) reproductive tract physiology. We recently showed that eCBs are produced differentially in the brain across the hormonal cycle in the rat [15], supporting the hypothesis that the production of all three eCB ligands is regulated by changes in the hormonal milieu. This hypothesis was suggested earlier when the presence of an estrogen responsive element was identified in the promoter region FAAH [16]. Additional support for this hypothesis was that Xiao and colleagues [17] showed that FAAH was regulated in the rat uterus across the hormonal cycle.

**Figure 1:**
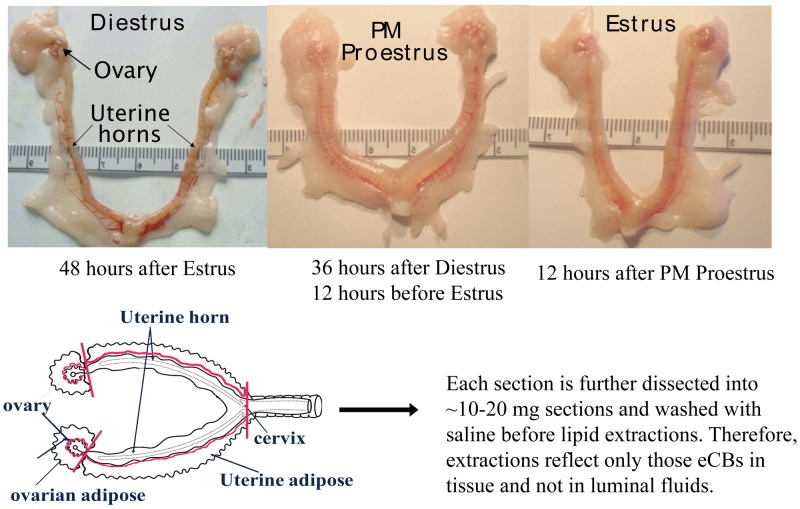
Female rat reproductive tract across the hormonal cycle and plan for dissection of tissue. Reproductive tract physiology changes dramatically across the hormonal (estrous) cycle of the rat. The bicornate uterine horns are inflamed on the evening of proestrus (PM proestrus) during sexual receptivity. In the absence of a copulatory signal, the uterine horns undergo a reduction in luminal fluid retention and size by the morning of Estrus. To determine the production of eCB ligands during these changes the reproductive tract was dissected into four gross tissue types: ovaries, ovarian adipose, uterine horns (referred after as uterus), and uterine adipose tissue.

In the current study, the eCBs AEA, 2-AG, and NAGly were measured in the female rat reproductive tract across the hormonal (estrous) cycle, in anovulatory retired breeders (RB), after ovariectomy (OVX) and with hormone (17β-estradiol and progesterone) replacement. The female rat reproductive tract has a bicornate uterus from which each horn is attached to an ovary via the fallopian tubes (Figure 1). As illustrated here, the uterine horns undergo dynamic changes throughout the hormonal cycle. Both the ovaries and the uterine horns are attached to specialized adipose tissue (hereafter referred to as ovarian fat and uterine fat). eCBs were measured in each of these four tissue types of the rat reproductive tract and were shown to be produced differentially by tissue type, hormonal and life cycle (*e.g.* age-related changes), and by exogenous changes in the hormonal milieu.

## 2. Results and Discussion

Each eCB was detected in each tissue examined. Measurements of eCBs at five time points of the hormonal cycle, in retired breeders, in ovariectomized (OVX), OVX +E2, OVX +P4, and OVX E2+P4 provides a comparison of ten distinct experimental groups for uterine tissues and 6 for ovarian tissues with an inherently complex set of interactions. To aid in interpretations, the data are presented individually as the organ first, the surrounding adipose tissue, then a combination of the two. Within each set of data statistical comparisons are first made between hormonal cycle groups, cycle data to retired breeders, cycle data to OVX, cycle data to OVX plus hormone replacement, and finally among the OVX and hormone replacement groups.

### 2.1. eCB Levels in Ovary and Ovarian Adipose across the estrous cycle and in retired breeders

**Figure 2:**
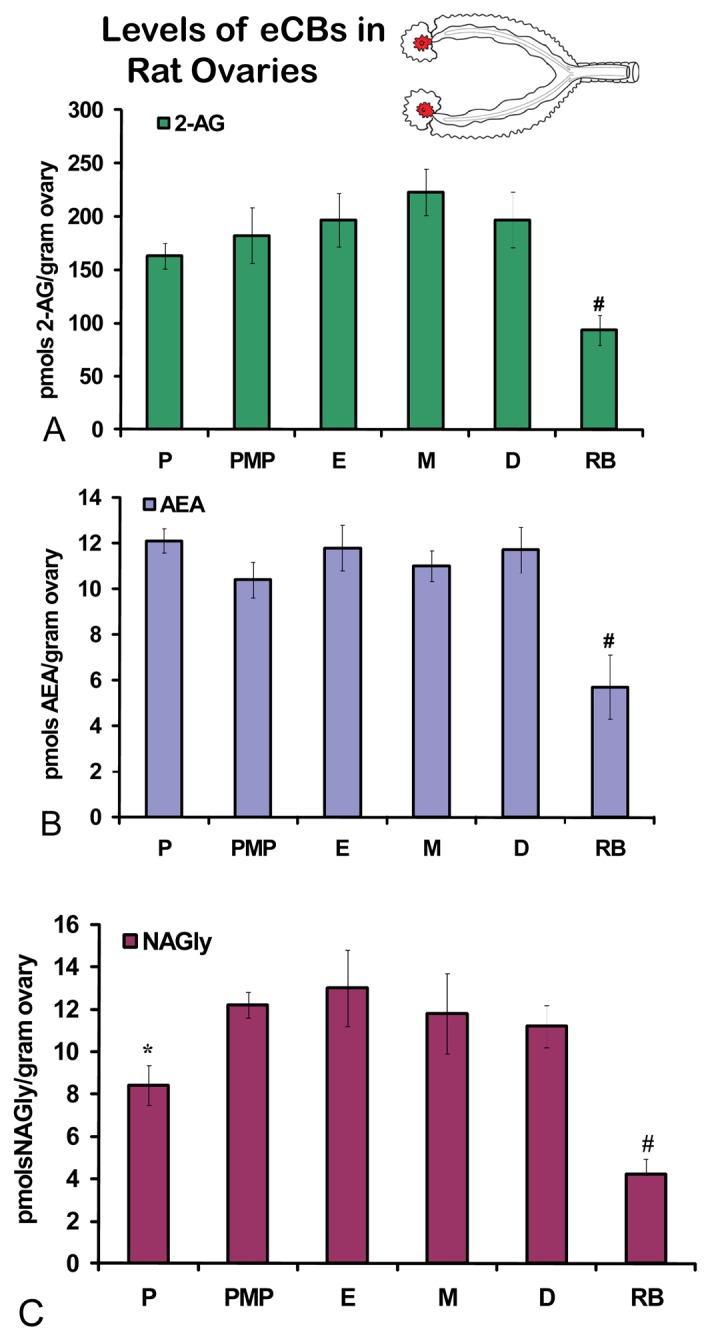
Levels of eCBs in rat ovaries across the hormonal cycle and in anovulatory retired breeders. P = proestrus AM; PMP = proestrus PM; E = estrus; M = metestrus, D = diestrus; RB = anovulatory retired breeders. * p ≤ 0.05 and represents comparisons across the hormonal cycle. # p ≤ 0.05 and represents comparisons of hormonal cycle groups with retired breeders.

**Figure 3:**
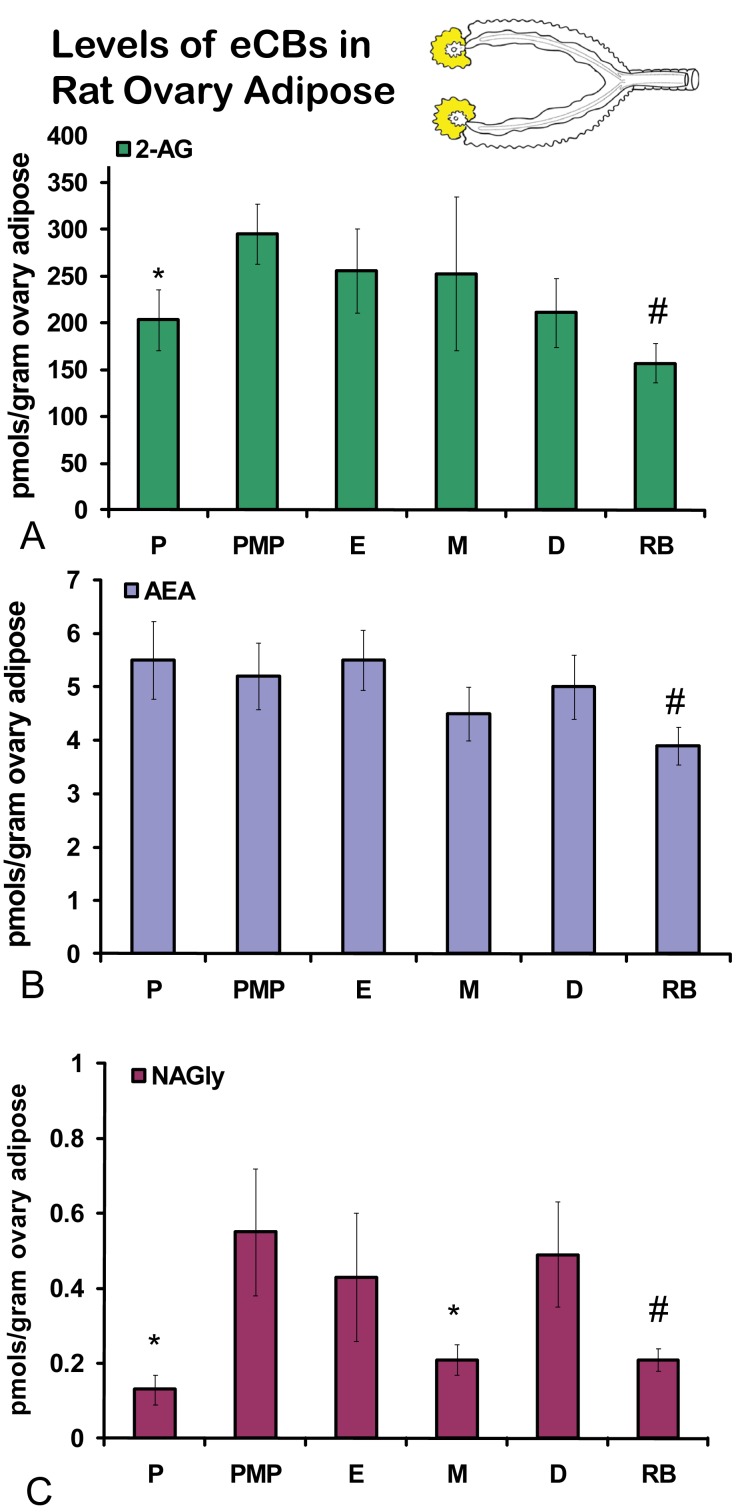
Levels of eCBs in rat ovarian adipose across the hormonal cycle and in anovulatory retired breeders. P = proestrus AM; PMP = proestrus PM; E = estrus; M = metestrus, D = diestrus; RB = anovulatory retired breeders. * p ≤ 0.05 and represents comparisons across the hormonal cycle. # p ≤ 0.05 and represents comparisons of hormonal cycle groups with retired breeders.

**Figure 4:**
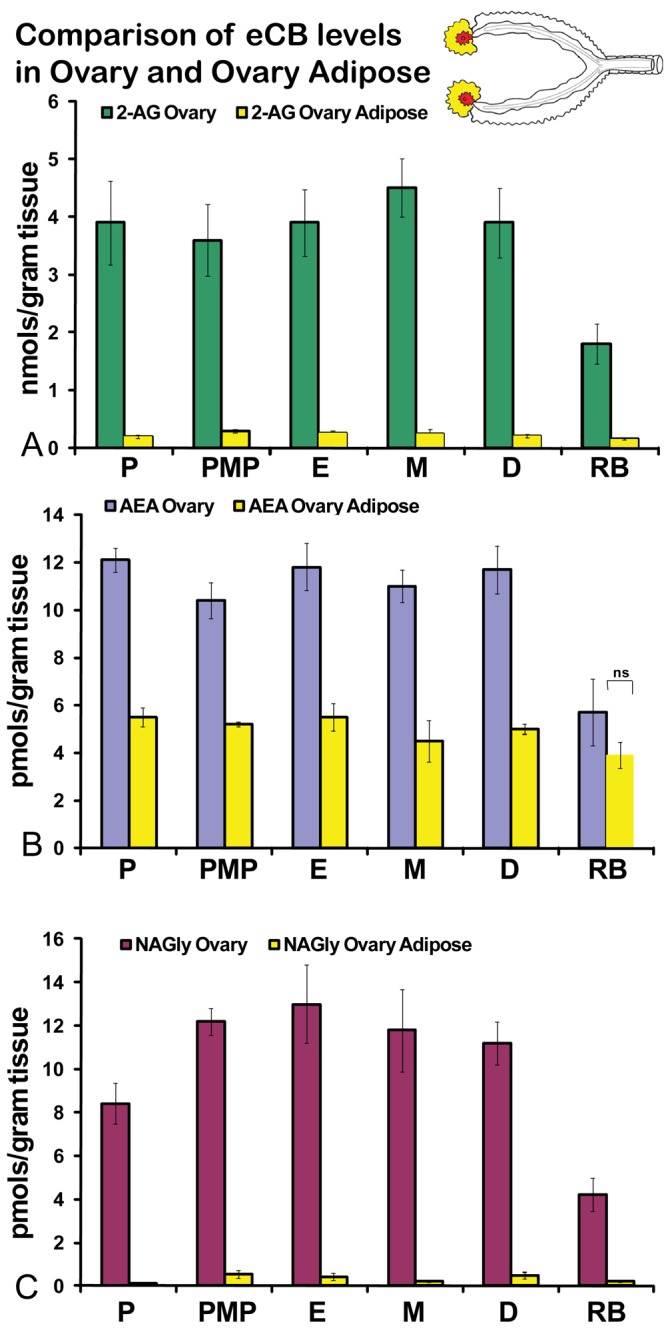
Comparisons of levels of eCBs in ovaries and ovarian adipose tissue. P = proestrus AM; PMP = proestrus PM; E = estrus; M = metestrus, D = diestrus; RB = anovulatory retired breeders. All experimental groups had significantly higher levels of eCBs in the ovary compared to the ovarian adipose tissue where p ≤ 0.05. The exception is labeled as NS for *not significant*.

No significant differences were measured in AEA or 2-AG levels in ovaries across the hormonal cycle (Figures 2A, B). In contrast, levels of NAGly were significantly lower in proestrus compared to the rest of the cycle (Figure 2C). All eCBs were significantly lower in the ovaries of anovulatory retired breeders compared to all days of the cycle (Figures 2A-C). In ovarian adipose tissue, 2-AG levels were significantly lower in proestrus compared to PM proestrus (Figure 3A). Similarly, NAGly levels were significantly lower in proestrus and metestrus compared to PM proestrus, estrus, and diestrus. The levels measured in the ovaries of all eCBs were significantly lower in the ovarian adipose of retired breeders compared to all days of the cycle (Figures 3A-C). Comparisons of eCB levels in ovaries and ovarian adipose showed that all eCBs were significantly lower in adipose tissue compared to the ovary with the exception of the levels of AEA in retired breeders, which were equivalent (Figure 4B).

### 2.2. Changes in uterine levels of eCBs across the hormonal cycle

**Figure 5:**
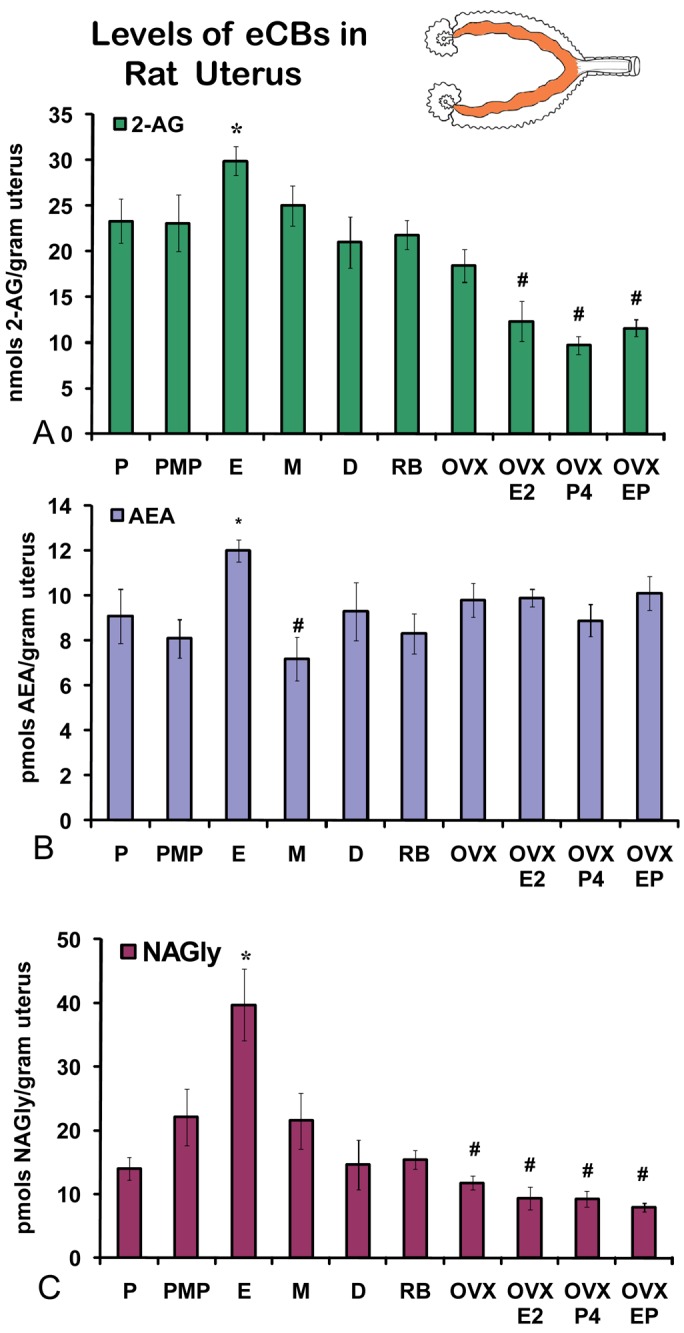
Levels of eCBs in rat uterus across the hormonal cycle, in anovulatory retired breeders (RB), after ovariectomy and hormone replacement. P = proestrus AM; PMP = proestrus PM; E = estrus; M = metestrus, D = diestrus; RB = anovulatory retired breeders; OVX = ovariectomy; E2 = 17βestradiol; P4 = progesterone. * p ≤ 0.05 and represents comparisons across the hormonal cycle. # p ≤ 0.05 and represents comparisons of hormonal cycle groups with retired breeders and OVX treatment groups.

**Figure 6:**
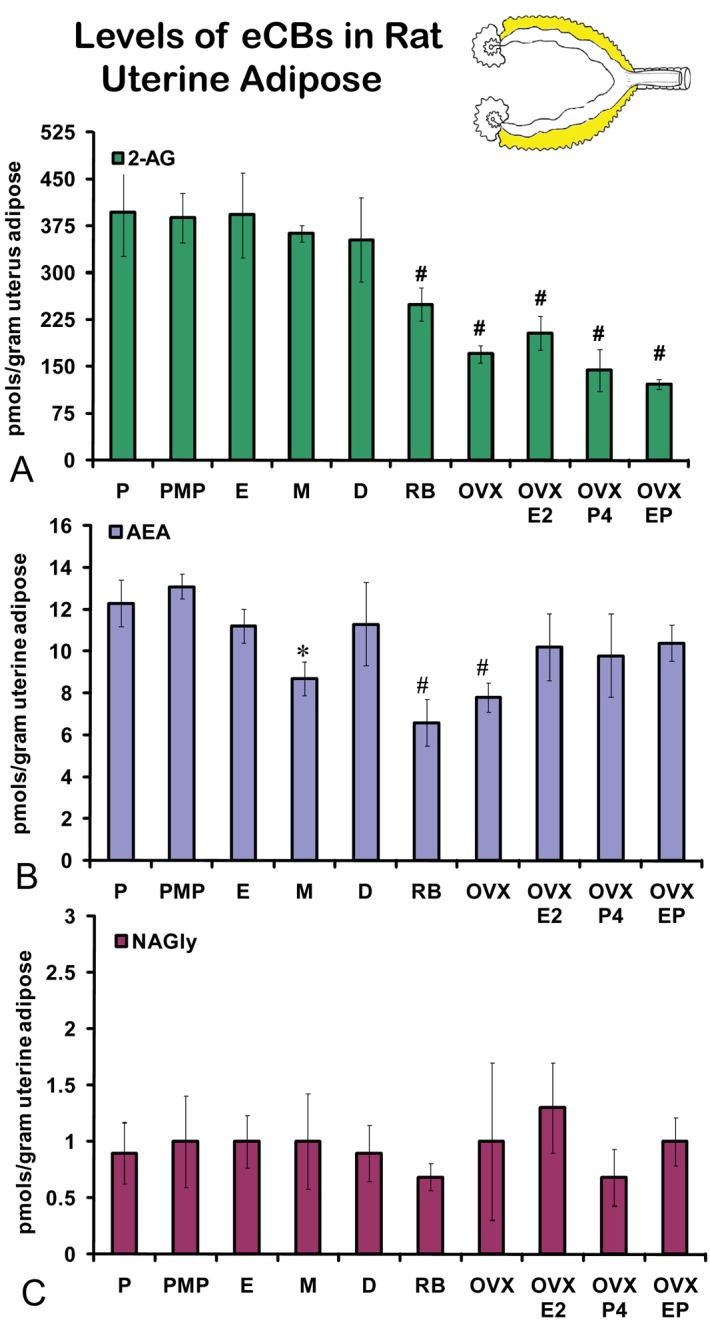
Levels of eCBs in rat uterine adipose tissue across the hormonal cycle, in anovulatory retired breeders (RB), after ovariectomy and hormone replacement. P = proestrus AM; PMP = proestrus PM; E = estrus; M = metestrus, D = diestrus; RB = anovulatory retired breeders; OVX = ovariectomy; E2 = 17βestradiol; P4 = progesterone. * p ≤ 0.05 and represents comparisons across the hormonal cycle. # p ≤ 0.05 and represents comparisons of hormonal cycle groups with retired breeders and OVX treatment groups.

**Figure 7:**
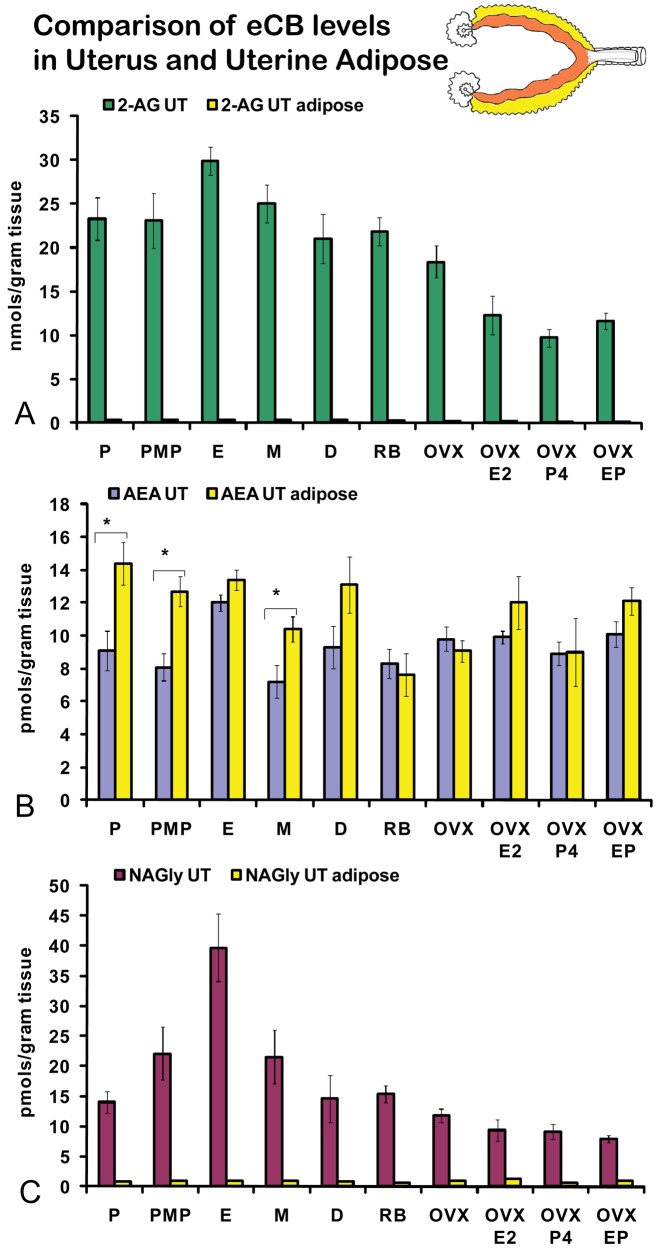
Comparisons of levels of eCBs in uterus and uterine adipose tissue. P = proestrus AM; PMP = proestrus PM; E = estrus; M = metestrus, D = diestrus; RB = anovulatory retired breeders; OVX = ovariectomy; E2 = 17βestradiol; P4 = progesterone. * p ≤ 0.05 and represents comparisons across the hormonal cycle. All experimental groups had significantly higher levels of 2-AG and NAGly in the uterus compared to the uterine adipose tissue where p ≤ 0.05. *p ≤ 0.05 in comparisons of AEA between the tissue types.

On the morning of estrus, all eCBs measured in the uterus were produced in significantly higher amounts than in any other stage of the hormonal cycle examined (Figures 5A-C). Changes in the levels of NAGly were greatest, doubling during the morning of estrus (Figure 5C). Uterine adipose tissue had relatively higher amounts of AEA compared to 2-AG and NAGly throughout the cycle and there were no differences across the cycle in levels of 2-AG and NAGly. Levels of uterine adipose AEA were significantly lower in metestrus (24 hour post estrus) compared to proestrus (AM and PM) and estrus (Figure 6B). Uniquely, levels of AEA were significantly higher in uterine adipose tissue than those produced in the uterus at three time points in the hormonal cycle reaching equivalence only during estrus (Figure 7B).

### 2.3. Changes in uterine levels of eCBs in retired breeders and with hormone replacement after ovariectomy (OVX)

Unlike the ovaries of retired breeders, the levels of eCBs in the retired breeder uterus were equivalent to those of all stages of the hormonal cycle except estrus (Figures 5 A-C). Similarly, levels of 2-AG after OVX were equivalent to those across the cycle with the exception of estrus. AEA levels after OVX were significantly higher than those in metestrus, whereas, levels of NAGly after OVX were significantly lower than those of in all cycle stages except diestrus (Figure 5C). Each hormone replacement group had a similar effect on the levels of 2-AG and NAGly in that the levels were significantly lower in these groups compared to all stages of the hormonal cycle, retired breeders, and OVX (Figures 5A,C). By contrast levels of AEA in the hormone replacement groups remained at equivalent levels to proestrus (AM and PM), diestrus, retired breeders, and OVX with the exception that levels of AEA in metestrus, were likewise, significantly lower than in all the hormone replacement groups.

Levels of 2-AG in uterine adipose tissue did not change across the hormonal cycle. However, the levels in retired breeders, OVX, and all hormone replacement groups were significantly lower than all stages of the hormonal cycle (Figure 6A). No change was measured in uterine adipose NAGly levels in any hormonal manipulation group (Figure 6C). Again, by contrast the levels of AEA in uterine adipose like those in ovarian adipose were relatively higher than 2-AG and NAGly. Showing more of a similar trend, the levels of AEA in metestrus were significantly lower than any stage of the hormonal cycle (like those of the uterus; Figure 6B). Similar to 2-AG, however, the levels of AEA in uterine adipose were significantly lower in retired breeders and after OVX. In contrast, the levels of AEA increased to equivalent levels of the hormonal cycle in all hormone replacement groups (Figure 6B).

Like ovarian adipose tissue, levels of 2-AG and NAGly measured in the uterine adipose tissue were significantly lower than those measured in the uterus in all experimental groups (Figures 7A, C). By contrast, levels of AEA in the uterine adipose were most often equivalent to those measured in the uterus within an experimental group and in three groups (AM and PM proestrus and diestrus) the levels of AEA in uterine adipose were higher than that measured in the uterus.

### 2.4. Discussion

Cannabis has been used by women for therapeutic purposes for a variety of ailments for millennia, with pelvic pain complaints ranking among the top uses [18]. Much of our current understanding of the eCB system in reproduction is involved in implantation and pregnancy. Thus, there is a need for a deeper understanding of the eCB system in the non-pregnant female reproductive tract.

Data about the eCB system in the larger realm of reproduction show that AEA decreases serum LH and prolactin in rats of both sexes [19]. When administered chronically in mice, AEA prolongs the duration of pregnancy, increases the rate of stillbirth, and temporarily inhibits postnatal development of offspring [19]. In humans, AEA concentrations were found in placenta, fetal membrane, umbilical vein, and umbilical artery and plasma from maternal circulation [20,21] further implicating endogenous cannabinoids in reproduction. In rats, the tissue levels of AEA did not correlate with plasma, suggesting that during pregnancy, maternal tissue levels of endocannabinoids are primarily regulated by *in situ* production and degradation to create endocannabinoid gradients conducive to successful pregnancy [21,22]. The data provided here are also demonstrate that cyclic and hormonal regulation of eCBs in the reproductive tract are region specific and that plasma levels are not a direct indicator of tissue levels.

Activity of fatty acid amide hydrolase (FAAH), the principal catabolic enzyme for AEA, has also been observed using radiochromatographic methods in the mouse uterus [23]. FAAH degrades AEA to ethanolamine and arachidonic acid (AA) [24]. In the uterus FAAH is localized in the endometrial epithelium, and it appears that sex hormones down-regulate FAAH activity and expression in early pregnancy. In rats, the activation and initiation of implantation requires higher expression of the FAAH gene in stromal cells and myometrial cells, indicating that the expression of FAAH mRNA is different in non-pregnant versus pregnant uterus [17]. In human endometrial biopsies immunoreactive FAAH staining observed in the glands of the menstrual phase is reduced to a significant degree in the early-proliferative phase. Glandular FAAH immunoreactivity remains low throughout the cycle reaching a lowest point in the mid-secretory phase [25]. Low FAAH in circulating maternal lymphocytes has been shown to be an early (<8 weeks of gestation) predictor of spontaneous abortion in humans ( [26]). Progesterone and leptin, alone or synergistically, upregulate FAAH resulting in reduced blood levels of AEA [26] and causing the CB1-dependent block of the release of leukemia inhibitory factor (LIF) to be removed. LIF is necessary for embryo implantation and survival [27]. Data presented here suggest that FAAH likely plays a primary role in regulating the levels of uterine eCBs. Future studies will be aimed at understanding the regulatory roles of steroid hormones on FAAH expression and activity.

CB1 mRNA has also been detected in pregnant or ovariectomized mouse uterus, both by Northern blot analysis and by reverse transcription coupled to the polymerase chain reaction [28], as well as in humans [29]. CB1 and CB2 receptor mRNAs are expressed in the ectoplacental cone and in the outer longitudinal layer and the inner circular layer of myometrial smooth muscles of the uterus of pregnant rat at gestational day eight, using immunohistochemical techniques [30]. In humans, CB1 protein was localized using immunohistochemical techniques in the smooth muscle of the wall, the smooth muscle of the endothelial vessels and luminal epithelium of the Fallopian tube [29]. In human endometrial biopsies, CB1 immunoreactivity is more intense in the glandular epithelium compared to that of the stroma and its expression in the glands is not regulated through the menstrual cycle [25]. Immunoreactive CB2 is observed in both the glands and stroma; this was minimal at the beginning of the cycle and increased with the most intense staining being observed during the late proliferative phase. During the secretory phase, CB2 immunoreactivity in the glands significantly reduced from the intense levels seen in the late proliferative phase reaching a nadir during the mid-secretory phase [31]. The current data demonstrating the production of NAGly in the female reproductive tract implicates GPR18 function as well and future studies will be aimed at characterizing this protein throughout the reproductive tract.

## 3. Experimental

### 3.1. Chemicals and Reagents

Progesterone (P4) and 17β-estradiol (E2), HPLC methanol, and ammonium acetate were purchased from Sigma Aldrich. Anandamide (AEA), 2-arachidonoylglycerol (2-AG), and *N*-arachidonoyl-glycine (NAGly) were purchased from Enzo Life Sciences. Deuterium-labeled AEA was purchased from Cayman Chemical. Solid phase extraction columns were purchased from Varian. HPLC water was obtained using a Millipore Ultrapure in-house filtration system.

### 3.2. Animal care and tissue collection

Female Sprague-Dawley rats were housed 3-4 per cage, maintained on a 12:12 light dark cycle, and fed and watered ad libitum. Hormonal (estrous) cycle was monitored by daily vaginal smear. Only those animals that maintained three consecutive 4-day estrous cycles were used for the study [32]. Hormonal/estrous phases were defined as Estrus; Metestrus (Estrus +24 hours); Diestrus (Estrus +48 hours); Proestrus (Estrus +72 hours); Proestrus PM (12 before Estrus).

Ovariectomies (OVX) were performed as previously described by the author [33] and the hormone replacement protocol of Xiao and colleagues [17]. In brief, two weeks after OVX animals were injected animals were injected with one of the following: sesame seed oil (0.1 mL/rat), E2 (0.1 mL/rat), P4 (0.4 mL/rat), or E2 + P4 (0.4 mL/rat). All steroids were dissolved in sesame oil and injected subcutaneously in the right thigh. Retired breeders were defined as those breeders who had ceased to have an estrous cycle and ceased to breed for at least one month were donated by the animal facility.

Rats were killed by rapid cervical dislocation 24 hours after injection and the reproductive tract removed and dissected as shown above (Figure 1). In brief, the complete reproductive tract was removed from the animal and placed on an ice-cold dissection plate. The uterine adipose tissue was first removed proximal to the uterine perimetrium, placed into an Eppendorf tube and flash frozen in liquid nitrogen. Then the uterus was dissected away from the ovaries at the distal end of the fallopian tubes and further dissected into ∼1 cm sections, allowing intra-luminal fluid to disperse. These sections were then gently washed with 0.9% sterile saline, and then transferred to an Eppendorf tube and flash frozen in liquid nitrogen. Ovarian tissue was taken in the same sequence: adipose tissue first followed by the ovary. Each was flash frozen in liquid nitrogen. All tissues were stored in -80C until used for lipid extractions.

### 3.3. Lipid extraction

Lipids were extracted as previously described by the author [15,34]. In brief, tissue was removed from -80 °C storage, weighed, and added directed to 40 volumes of HPLC methanol. One hundred pmol of deuterium-labeled AEA was spiked to the sample at this time. Samples were then homogenized using a Polytron for 1-2 minutes while being submerged into an isopropanol-ice slurry to avoid heating. Samples were then centrifuged at 19,000 × g for 20 minutes at 24C. The supernatant was then transferred to fresh tubes and HPLC water added to make a 25% organic solution.

Partial purification was achieved by C18 solid phase extraction columns as previously described [15]. In brief, columns were activated with 2.5 mL 100% HPLC methanol, 1.5 mL HPLC water and then loaded with the 25% organic supernatant. Columns were then washed with 2.5 mL HPLC water, followed by elutions of 40, 65, 85, and 100% HPLC methanol.

### 3.4. HPLC/MS/MS analysis

Rapid separation of analytes was obtained using 10 μL injections of analyte (Shimadzu system autosampler) onto a Zorbax eclipse XDB 2.1 × 50 mm reversed phase column. Gradient elution (200 μL/min) was performed under pressure on a pair of Shimadzu (Columbia, Maryland) 10AdVP pumps. Mass spectrometric analysis was performed with an Applied Biosystems/MDS Sciex (Foster City, CA) API 3000 triple quadrupole mass spectrometer equipped with a heat-assisted electrospray ionization source. Mobile phase for the positive ion mode was a gradient presentation of mobile phase A: 20% HPLC-grade methanol (80% HPLC-grade H_2_O), 1 mM ammonium acetate, 0.5% acetic acid, and mobile phase B: 100% HPLC-grade methanol, 1 mM ammonium acetate, 0.5% acetic acid. Mobile phase for negative ion mode analysis (NAGly detection) was A: 20% HPLC-grade methanol (80% HPLC-grade H_2_O), 1 mM ammonium acetate, and mobile phase B: 100% HPLC-grade methanol, 1 mM ammonium acetate. Levels of each compound were analyzed by multiple reactions monitoring (MRM) on the LC/MS/MS system. In MRM mode, detection of each compound is based on fragmentation of the precursor ion [M +H]^+^ or [M-H]^+^ to yield a prominent product ion. Massspectrometric conditions were optimized for each compound using direct flow injection of synthetic standards of each compound. The molecular ion and fragment (MI/F) for each compound measured were as follows for positive ion mode: 2-AG 379.3/287.3; AEA 348.3/287.3; for negative ion mode: NAGly 360.3/74.2. 2-AG and AEA were recovered in the 100% HPLC methanol elution, whereas, NAGly was recovered in the 85% HPLC methanol elution.

### 3.5. Data Analysis

Each tissue type was processed at the same time and analyzed continuously using HPLC/MS/MS. This allows for the use of the same standard curve, mobile phase, and analytical column. This methodology greatly reduces between subject variability. Likewise, the adipose tissue associated with the organ was analyzed in sequence with the previous organ to allow for the closest comparison between these groups as well. Each sample was spiked with deuterium-labeled AEA at the beginning of the extraction process to standardize the level of extraction for eCBs in each tissue. The amounts of moles per injection were determined comparing standard curves of each analyte using Analyst 4.2 software (Applied Biosystems/MDS Sciex). Levels of moles/gram were determined by dividing the number of total moles in the eluant by the total tissue weight in grams. All data were first analyzed using ANOVA with a post-hoc Fisher's least significant difference test. Significance was set as p ≤ 0.05.

## 4. Conclusions

Data presented here show that the eCBs, AEA, 2-AG, and NAGly are dynamically and individually regulated in the rat reproductive tract by changes in the hormonal milieu. These data also suggest a novel function for adipose tissue associated with aspects of the female reproductive tract, namely, that adipose tissue may also be responsive to changes in the hormonal environment and that it may act in a signaling capacity and not only for energy storage and encasement of the organ.
